# A Prospective Study to Investigate Controlling Blood Pressure Under Transcranial Doppler After Endovascular Treatment in Patients With Occlusion of Anterior Circulation

**DOI:** 10.3389/fneur.2021.735758

**Published:** 2021-09-29

**Authors:** Chunrong Tao, Pengfei Xu, Yang Yao, Yajuan Zhu, Rui Li, Jie Li, Wenwu Luo, Wei Hu

**Affiliations:** ^1^Stroke Center & Department of Neurology, The First Affiliated Hospital of USTC, Division of Life Sciences and Medicine, University of Science and Technology of China, Hefei, China; ^2^Department of Neurosurgery, The First Affiliated Hospital of USTC, Division of Life Sciences and Medicine, University of Science and Technology of China, Hefei, China; ^3^Department of Ultrasound, The First Affiliated Hospital of USTC, Division of Life Sciences and Medicine, University of Science and Technology of China, Hefei, China; ^4^Department of pathology, The First Affiliated Hospital of Anhui Medical University, Hefei, China

**Keywords:** cerebral blood flow, TCD, endovascular thrombectomy, stroke, occlusion in anterior circulation

## Abstract

**Objective:** The objective of this study was to evaluate the effect of blood pressure (BP) management with transcranial Doppler (TCD) guidance in patients with large-vessel occlusion in the anterior circulation after endovascular thrombectomy (EVT) on the long-term prognosis.

**Methods:** This was a prospective study; 232 patients were nonrandomized assigned to TCD-guided BP management (TBM) group or non-TCD-guided BP management (NBM) group. In the TBM group, BP was controlled according to TCD showing cerebral blood flow fluctuation. In the NBM group, BP was controlled according to the guidelines. The primary endpoint was a modified Rankin scale (mRS) score of 2 or lower at 90 days. The safety outcomes were the rates of symptomatic or any intracerebral hemorrhage (ICH) and mortality at 90 days.

**Results:** One hundred sixty-three patients were assigned to the TBM group, and 69 were assigned to the NBM group. In the propensity score-matched cohort (65 matches in both groups), there was significant difference in the proportion of participants with mRS 0–2 at 90 days according to BP management (adjusted odds ratio 3.34, 95% CI 1.36 to 8.22). There was no difference in the rates of symptomatic or any ICH and mortality between two groups. In inverse probability-weighted regression adjustment analysis, mortality decreased significantly in the TBM group than in the NBM group (adjusted odds ratio 0.86, 95% CI 0.76–0.99, *p* = 0.03).

**Conclusion:** In patients with acute ischemic stroke from large-vessel occlusion in the anterior circulation, BP management under TCD was superior to NBM in improving the clinical outcomes at 90 days.

**Clinical Trial Registration:** (URL: https://www.chictr.org.cn/showproj.aspx?proj=55484; Identifier: ChiCTR2000034443.

## Introduction

Although endovascular treatment (EVT) is the most effective treatment for cerebral large-vessel occlusion (LVO) of anterior circulation (1–5), <50% of patients can achieve functional independence in activities of daily living after EVT (6), which was partly due to cerebral hemodynamic abnormalities after AIS.

There is still no consensus with respect to the optimal scheme of blood pressure (BP) management in AIS patients after EVT. Studies of the association between postoperative BP and clinical outcomes suggested that postoperative BP management is interacted with recanalization status in which successful recanalization needs lower BP to decrease the risk of brain edema and intracranial hemorrhage (ICH), while non-recanalization needs higher BP to sustain collateral perfusion and decrease final infarction volume. After intravenous thrombolysis (IVT), <30 to 40% of the patients with proximal arterial occlusions could achieve successful recanalization, while a successful recanalization can be achieved in about 70 to 80% of AIS patients after EVT (7).

Recently, several studies attempted to explore whether abnormal cerebral blood flow under transcranial Doppler (TCD) can predict the long-term prognosis (8–11). TCD can monitor the cerebral blood flow changes in the early postinterventional period, reflect changes in cerebral hemodynamics in real time, and directly assess the change in intracranial pressure, making TCD an option for evaluating cerebral perfusion constantly. TCD can be performed bedside with dynamic repetition, which has a high agreement with digital subtraction angiography (DSA). A prospective randomized controlled study including 95 patients showed a significantly lower incidences of early neurological deterioration and 3-month mortality in patients with TCD-guided cerebral blood flow management than patients with fixed static systolic BP levels; however, insufficient sample size decreased the robustness of the results and no conclusion about the 3-month's functional outcome was drawn (12).

We hypothesized that precise management of BP according to individual cerebral blood flow fluctuations with TCD monitoring will improve efficacy and safety outcomes in AIS patients from LVO in the anterior circulation.

## Methods

### Participants

This study included consecutive patients with occlusion of the intracranial internal carotid artery (ICA) and proximal middle cerebral artery (MCA) who received EVT between January 2017 and June 2020. This was a nonrandomized, interventional study, where consecutive patients were enrolled prospectively. The patients were assigned to either TCD-guided BP management (TBM) group or a non-TCD-guided BP management (NBM) group. The choice was determined by the clinicians and by the patients’ preference.

The study protocol was approved by the Medical Ethics Committee of the First Affiliated Hospital of USTC. Written informed consent was given by all patients or their relatives before participation.

### Eligibility Criteria

The inclusion criteria were as follows: age ≥18 years; time from onset to puncture within 24 h after symptom onset. The exclusion criteria were as follows: known thrombocytopenia at presentation or a thrombocyte count of 100× 10^9^/L or lower; ICH; malignant edema; renal insufficiency (creatinine clearance rate <30 ml/min); hepatic dysfunction (serum alanine transaminase >twice the upper limit of the normal value, or serum aspartate transaminase >twice the upper limit of the normal value); pregnant women; premorbid mRS>2; recent major bleedings, surgery, or trauma; difficulty in detecting the acoustic window by TCD; and complete occlusion of MCA on the lesion side prevented cerebral blood flow parameters from being obtained. All including patients treated with standard, up-to-date EVT protocols.

### Brain Imaging

After the first cerebral computed tomography (CT) to exclude cerebral hemorrhage or diagnosis unrelated to AIS, a second cerebral CT was performed 246 ± h postoperation.

### TCD Monitoring Scheme

Patients in the TBM group received bedside TCD monitoring for at least 24 h after EVT (EMS-9D; Delica, Shenzhen, China). All TCD operations were performed by one qualified operator. Bilateral MCAs were selected as the monitoring artery in this study, sampling volume was 12–15mm, and the monitoring depth was 50–60 mm (13). TCD sonography with 2-MHz ultrasound probes was applied to measure end diastolic velocity (EDV) and peak systolic velocity (PSV). Direction of the blood flow and typical depth of the signal assisted in the definition of the signals of the MCAs. A mechanical probe holder was used to maintain a constant probe position.

Mean flow velocity (MFV) was calculated automatically using the following equation: MFV = (PSV – EDV) / 3 + EDV. PSV was selected as the primary target in this study because previous research showed that the risk of early neurological deterioration significantly increased when PSV ≤74 cm/s or ≥118 cm/s among patients after anterior circulation EVT (14). A continuous monitoring of MCA cerebral blood flow velocity was conducted, and an automatic reminder was set up when PSV ≥ 118 cm/s, MFV on the lesion side was higher than 125% that on the opposite side, and PSV ≤ 74 cm/s (14).

### BP Monitoring Scheme

All patients underwent cuff BP monitoring for at least 24 h after EVT.

### BP Management

In the TBM group, patients received BP-lowering treatment when the cerebral blood flow was accelerated (PSV ≥ 118 cm/s or MFV on the lesion side was higher than 125% that on the opposite side), and patients received BP-raising treatment when the cerebral blood flow was decelerated (PSV ≤ 74 cm/s) (14). All patients in the TBM group received BP intervention when TCD parameters became abnormal regardless of the BP level. In the NBM group, patients with successful reperfusion received BP-lowering treatment when systolic BP (SBP) > 140 mm, and patients with unsuccessful reperfusion received BP-lowering treatment when BP was >180/105 mm Hg.

**Figure 1:**
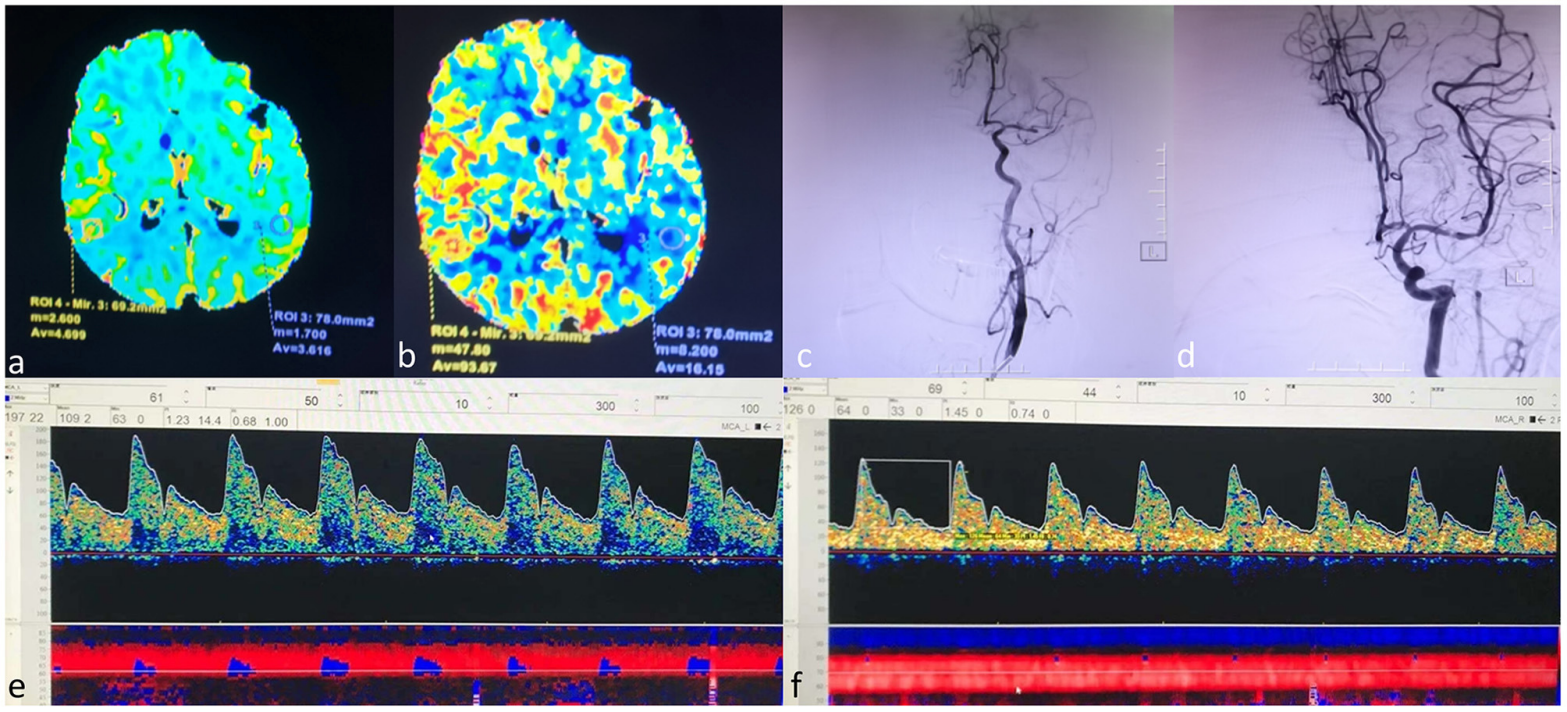
A case example. An 80-year-old female who received a successful mechanical thrombectomy in the left middle cerebral artery (MCA) branch M1. The CTP series showed that CBV did not increase **(a)**. Mildly increased CBF **(b)**. The left MCA occluded entirely on DSA before EVT **(c)**. The left MCA reappeared entirely on DSA after EVT **(d)**. The PSV of the left MCA was 197 cm/s, and MFV increased higher than 125% compared to that on the right side [**(e)**, left MCA; **(f)**, right MCA]. The patient was given nicardipine until the flow velocity of the left MCA was restored to the normal. The patient had no hemorrhagic transformation and achieved an mRS score of 0 at 90 days. MCA=middle cerebral artery, CTP=computed tomography perfusion, CBV=cerebral blood volume, CBF, cerebral blood flow, DSA, digital subtraction angiography, EVT, endovascular thrombectomy, PSV, peak systolic velocity, MFV, mean flow velocity.

Treatments for BP-lowering were administered as follows: the first-line antihypertensive medication was intravenous infusion of urapidil, and the second-line medication was intravenous infusion of diltiazem hydrochloride. Treatments for BP-raising were administered as follows: an intravenous rapid infusion of 0.9% sodium chloride solution (500 ml) and an intravenous pumping of dopamine or norepinephrine when needed. A case example is shown in Figure 1.

If BP increased or decreased significantly when applying BP treatment in the TBM group, such as when the systolic BP was lower than 90 mmHg or >180 mmHg, the BP lowering or raising treatment would be suspended.

### Data Collection and Assessment

Baseline demographic and clinical information for all enrolled patients were recorded, including age, sex, admission National Institutes of Health Stroke Scale (NIHSS) score, presence of hypertension, diabetes mellitus, hyperlipidemia, auricular fibrillation, smoking history, time from stroke onset to puncture, and time from puncture to the recanalization.

Our primary outcome was the excellent outcome, defined as a modified Rankin scale (mRS) score 0–2 at 90 days (±14 days). Ninety days after the acute event, functional outcome was assessed by board-certified vascular neurologists during a routinely scheduled clinical visit or by a study nurse certified in administering the mRS during a standardized telephone interview if the patient was unable to attend. Secondary outcomes included favorable outcome (mRS score 0–3) and the distribution of mRS score at 90 days (±14 days). Safety outcomes included the all-cause mortality at 90 days and the occurrence of cerebral hemorrhage according to the ECASS II (European Collaborative Acute Stroke Study) classification (15). Adverse events due to BP treatments mainly consisted of hypotension or hypoperfusion requiring fluid or vasopressor administration, with or without symptoms, with systolic BP of <80 mm Hg or PSV ≤74 cm/s were documented as nonserious adverse events.

### Statistical Analyses

Continuous variables were described as mean and SD, and categorical variables were presented as frequencies. Baseline characteristics were described according to the assigned group, and the absolute standardized difference (ASD) was used to assess the magnitude of the between-group differences; an ASD >10% could be interpreted as a meaningful difference (16). We compared the outcomes between the two study groups after taking into account the potential confounding factors by using propensity score methods (PSM) (17).

The effects of the treatment were estimated by using PSM as primary analysis and by using the inverse probability-weighted regression adjustment (IPWRA) model (inversed probability weights were obtained to calculate the outcome-regression parameters that account for the missing-data problem arising from the fact that each subject is observed in only one of the potential outcomes) as a secondary analysis.

A multivariable probit regression model was used to calculate the propensity score, and TCD monitoring was used as the dependent variable and all the obtained variables related to the outcomes as covariates. Patients in the NBM group were matched 1:1 to patients in the TBM group based on corresponding PSM, using the nearest neighbor matching algorithm with a caliper width of 0.2 of the propensity score. To evaluate bias reduction, ASDs were calculated again after PSM (18, 19). A forward stepwise method was used to determine the final multivariable model, and the significance level for addition to the model was set as 0.1.

In the IPWRA model, we used a model to predict treatment status and another model to predict outcomes. The between-group comparisons were done with a three-step approach. Firstly, the parameters of the treatment model and the inverse-probability weights were calculated. Secondly, weighted regression models of the outcome for each treatment level were fitted and the treatment-specific predicted outcomes for each subject with the estimated inverse-probability weights were obtained. Thirdly, the means of the treatment-specific predicted outcomes were calculated, and the contrasts of these averages provide the estimates of the average treatment effect on the treated (20).

Based on the multivariable analysis after PSM, we formulated nomograms to predict the risk of excellent and favorable outcome. Collinearity of combinations of variables was evaluated by the variation inflation factors (<2 being considered nonsignificant) and condition index (<30 being considered nonsignificant). The performance of the nomogram was assessed with a calibration curve and concordance index (C-index) to measure the internal calibration and discriminative ability. Bootstraps with 2,000 resamples were used. A C-statistic over 0.7 indicates a good prediction of functional outcomes.

Statistical testing was conducted at the two-tailed level of 0.05. All analyses were performed using STATA version 14.2, and the significance level was set at 0.05.

### Data Availability Statement

Anonymized data will be shared by request from any qualified investigator.

## Results

**Figure 2:**
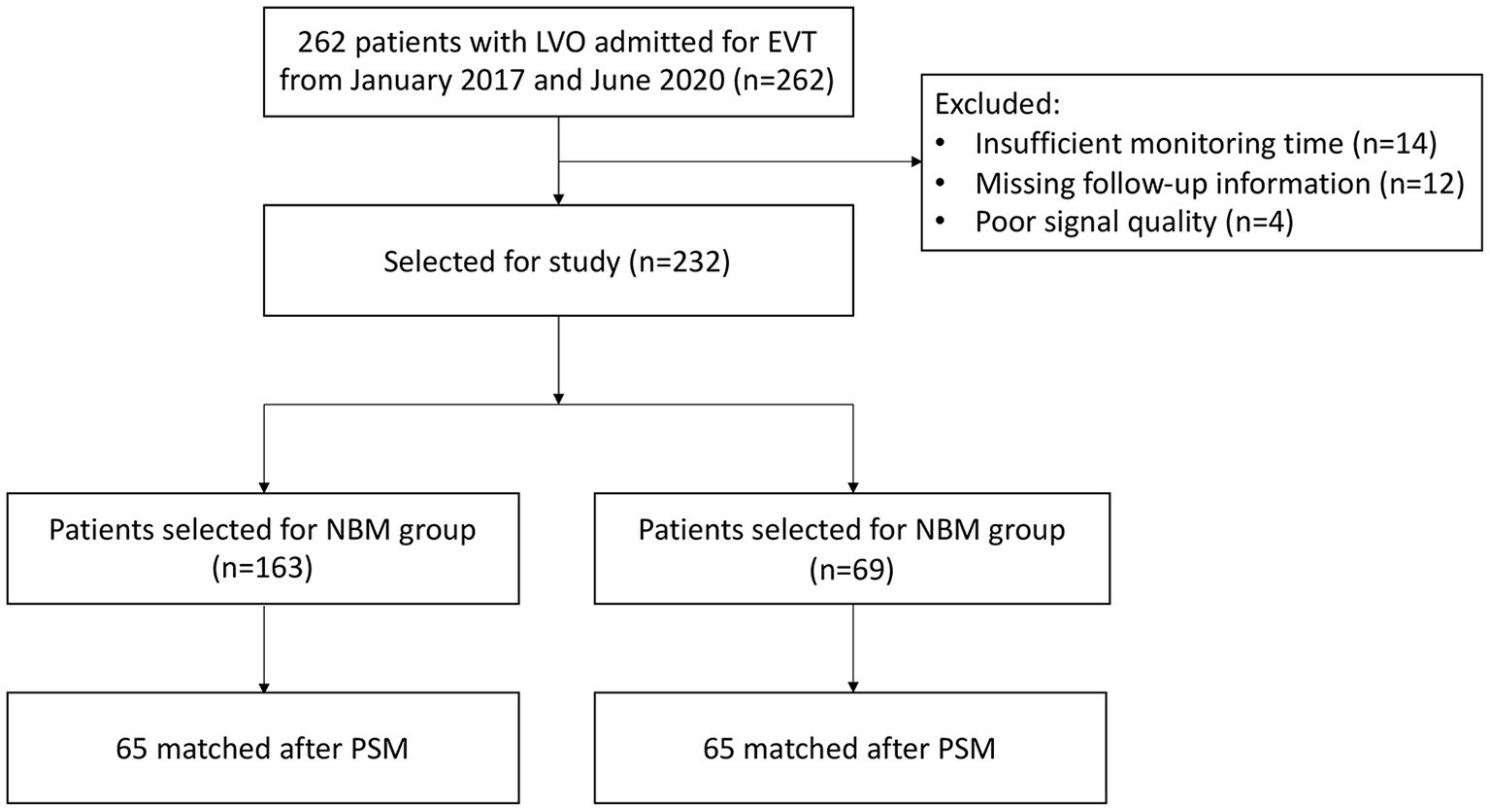
Study flowchart. LVO, large-vessel occlusion; NBM, non-TCD-guided BP management; TBM, TCD-guided BP management; PSM, propensity score methods.

Two hundred sixty-two patients were initially enrolled in the study; 30 patients were excluded from analysis due to insufficient monitoring time (*n* = 14), missing follow-up information (*n* = 12), and poor signal quality (*n* = 4). Thus, a total of 232 patients were left in the study (Figure 2). Of these, 69 patients were assigned to the TBM group and 163 patients were assigned to the NBM group. In the NBM group, 28 (17.2%) patients received BP-lowering treatment and 15 (9.2%) patients received BP-raising treatment. In the TBM group, 35 (50.7%) patients received BP-lowering treatment and 16 (23.2%) patients received BP-raising treatment. Three (1.8%) patients in the NBM group and two (2.9%) patients in the TBM group received dopamine or norepinephrine for the BP-raising. During the first 24 h postoperation, among 202 (87.1%) of 232 patients with consecutive SBP measurements and TCD measurements, the mean time spent in the assigned target range was 95.1% in the NBM group and 72.5% in the TCD group.

Sixty-five matched pairs were found in the primary analysis. Table 1 shows the baseline characteristics according to the two study groups before and after PSM. Before matching, age, admission SBP, admission diastolic blood pressure (DBP), baseline NIHSS, IVT, stroke etiology, history of hypertension, diabetes and AF, puncture to reperfusion time (PTR), and modified thrombolysis in cerebral infarction (mTICI) showed meaningful differences (ASD >10%). ASD reduced significantly after PSM with a maximum ASD of 1.9% for age, 0.7% for admission SBP, 5.7% for admission DBP, 16.5% for IVT, and 0 for history of HBP (Table 1). The percentage of patients with successful reperfusion (eTICI score, ≥2b) on final angiography was 100% in the NBM group and 98.5% in the TBM group, respectively. ASD remained significant after PSM for IVT use (16.5%), stroke etiology (31.8%), and auricular fibrillation (18.7%) and became significant for sex (16.2%) and occlusion sites (18.6%).

**Table 1:** Baseline clinical and anatomic characteristics, endovascular procedures, and clinical outcomes of patients with ELVO according to blood flow monitoring approach before and after PSM.

	**Before matching**			**After matching**		
	**NBM (***n =*** 163)**	**TBM (***n =*** 69)**	**ASD, %**	**NBM (***n =*** 65)**	**TBM (***n =*** 65)**	**ASD, %**
Age, mean (SD), year	66.7 (13.4)	65 (13.2)	12.8	65.9 (13.7)	65.6 (12.7)	1.9
Admission SBP, mean (SD), mm Hg	147.4 (21.3)	138.1 (22.3)	42.8	138.5 (14.1)	138.4 (22.4)	0.7
Admission DBP, mean (SD), mm Hg	86.1 (13.9)	82.4 (11.8)	29.1	83.1 (11.3)	82.4 (11.9)	5.7
Premorbid mRS score, mean (SD)	0.3 (0.9)	0.3 (1.1)	6.2	0.3 (1)	0.3 (1.1)	5.8
NIHSS score prior to treatment, mean (SD)	15.7 (9.4)	13.9 (7.3)	22	13.2 (8.3)	13.9 (7.4)	7.8
Female, *n* (%)	70 (42.9)	27 (39.1)	7.8	20 (30.8)	25 (38.5)	16.2
Tobacco use, *n* (%)	32 (19.6)	11 (15.9)	9.7	11 (16.9)	10 (15.4)	4.2
Prior use of IV thrombolysis, *n* (%)	29 (17.8)	9 (13)	13.2	13 (20)	9 (13.8)	16.5
Tirofiban use, *n* (%)	68 (41.7)	26 (37.7)	8.3	25 (38.5)	25 (38.5)	0
Occlusion site, *n* (%)						
ICA	81 (49.7)	34 (49.3)	0.8	36 (55.4)	32 (49.2)	12.3
MCA M1/M2	82 (50.3)	35 (50.7)		29 (44.6)	33 (50.8)	
Stroke etiology, *n* (%)						
LAA	88 (54)	43 (62.3)	37.1	42 (64.6)	41 (63.1)	31.8
Cardioembolic	67 (41.1)	18 (26.1)		21 (32.3)	17 (26.2)	
Undetermined etiology	8 (4.9)	8 (11.6)		2 (3.1)	7 (10.8)	
Medical history						
Hypertension, *n* (%)	98 (60.1)	38 (55.1)	10.2	36 (55.4)	36 (55.4)	0
Diabetes, *n* (%)	28 (17.2)	9 (13.0)	11.6	9 (13.8)	8 (12.3)	4.6
Auricular fibrillation, *n* (%)	63 (38.7)	32 (46.4)	15.7	25 (38.5)	31 (47.7)	18.7
Stoke or TIA, *n* (%)	27 (16.6)	13 (18.8)	6	13 (20)	12 (18.5)	3.9
Recanalization process and outcomes						
OTP, mean (SD), min	485.1 (249.1)	495.8 (278.2)	4.1	514.3 (266.6)	500.9 (284.7)	4.9
PTR, mean (SD), min	141.9 (57.6)	135.1 (45.9)	13	135 (45.8)	136.6 (45.5)	3.6
mTICI score 2b or 3, *n* (%)	155 (95.1)	68 (98.6)	19.8	65 (100)	65 (98.5)	17.7

*ASD, absolute standardized difference; SBP, systolic blood pressure; DBP, diastolic blood pressure; mRS, modified Rankin scale; NIHSS, National Institutes of Health Stroke Scale; IV, intravenous; ICA, intracranial carotid artery; MCA, middle cerebral artery; IQR, interquartile range; LAA, large-artery atherosclerosis; OTP, onset to groin puncture time; PTR, puncture to reperfusion time; mTICI, modified thrombolysis in cerebral infarction*.

**Figure 3:**
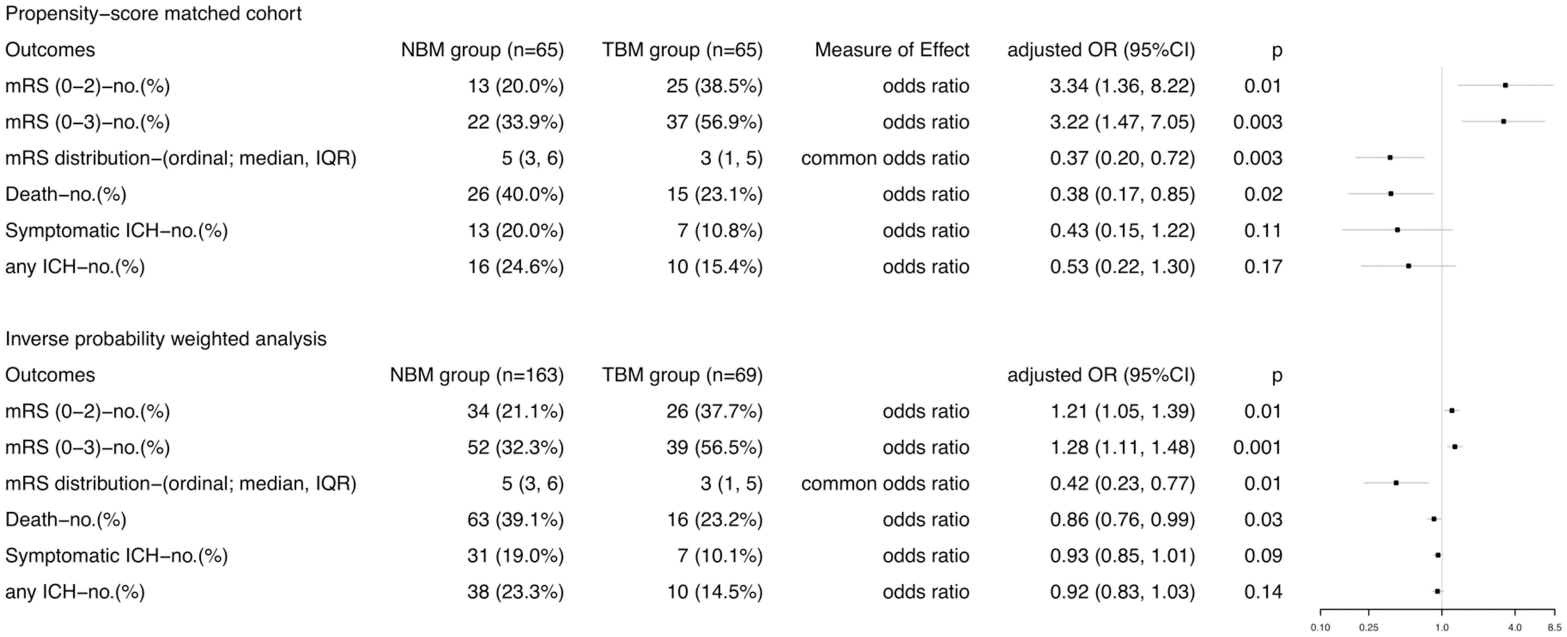
Comparisons in efficacy and safety outcomes according to BP management approach in stroke patients treated with thrombectomy in PSM and IPWRA analyses. Primary outcome measure was the excellent outcome, which was defined as 90-day mRS score 0-2. mRS, modified Rankin scale; ICH, intracranial hemorrhage.

After forward stepwise analyses, TCD use, baseline NIHSS, tirofiban use and onset to groin puncture time (OTP) remained independent predictors. After PSM, occlusion site and stroke etiology were still significantly different between the NBM and TBM groups; however, the multivariable analysis showed that they did not influence the results materially. In the PSM cohort (Figure 3), excellent outcome was significantly higher in the TBM group (38.5%) than in the NBM group (20%, adjusted OR [aOR] 3.34, 95% confidence interval [CI] 1.36–8.22, *p* = 0.01). Similarly, the rate of favorable outcome was achieved significantly more often in the TBM group than in the NBM group (aOR 3.22, 95% CI 1.47–7.05, *p* = 0.003). The adjusted common odds ratio (acOR) for the mRS score at 90 days was 0.37 (95% CI 0.20–0.72; *p* = 0.003). In the IPWRA model (Figure 3), significant results were found for excellent outcome analysis (aOR 1.21, 95% CI 1.05–1.39, *p* = 0.01), favorable analysis (aOR 1.28, 95% CI 1.11–1.48, *p* = 0.001), and mRS distribution analysis (acOR 0.42, 95% CI 0.23–0.77, *p* = 0.01).

Receiving sedation or anesthesia was associated with a more stable BP. With the IPWRA model, sensitivity analyses that restricted to cases with sedation or anesthesia (patients with sedation or anesthesia (*n* = 74): mRS 0–2 vs. mRS 3–6, OR: 1.23, 95% CI: 0.93–1.64; patients without sedation or anesthesia (*n* = 156): mRS 0–2 vs. mRS 3–6, OR: 1.20, 95% CI: 1.01–1.42) did not materially alter the results (p for interactio*n* = 0.61).

With respect to the safety outcomes, mortality at 90 days was 40.0% in the NBM group and 23.1% in the TBM group (aOR 0.43, 95% CI 0.15–1.22, *p* = 0.11). There was no difference of the percentages of patients with symptomatic or any ICH in the TBM group and in the NBM group (Figure 3). In the IPWRA model, no significant results were found for sICH and ICH (Figure 3); however, TCD monitoring BP control was significantly associated with decreased risk of mortality at 3 months (aOR 0.86, 95% CI 0.76–0.99, *p* = 0.03). No patients experienced hypotensive or hypoperfusion events due to BP treatment requiring fluid or vasopressor administration.

### Multicollinearity Test, Nomograms, and Validation of Predictive Accuracy for Efficacy

**Figure 4:**
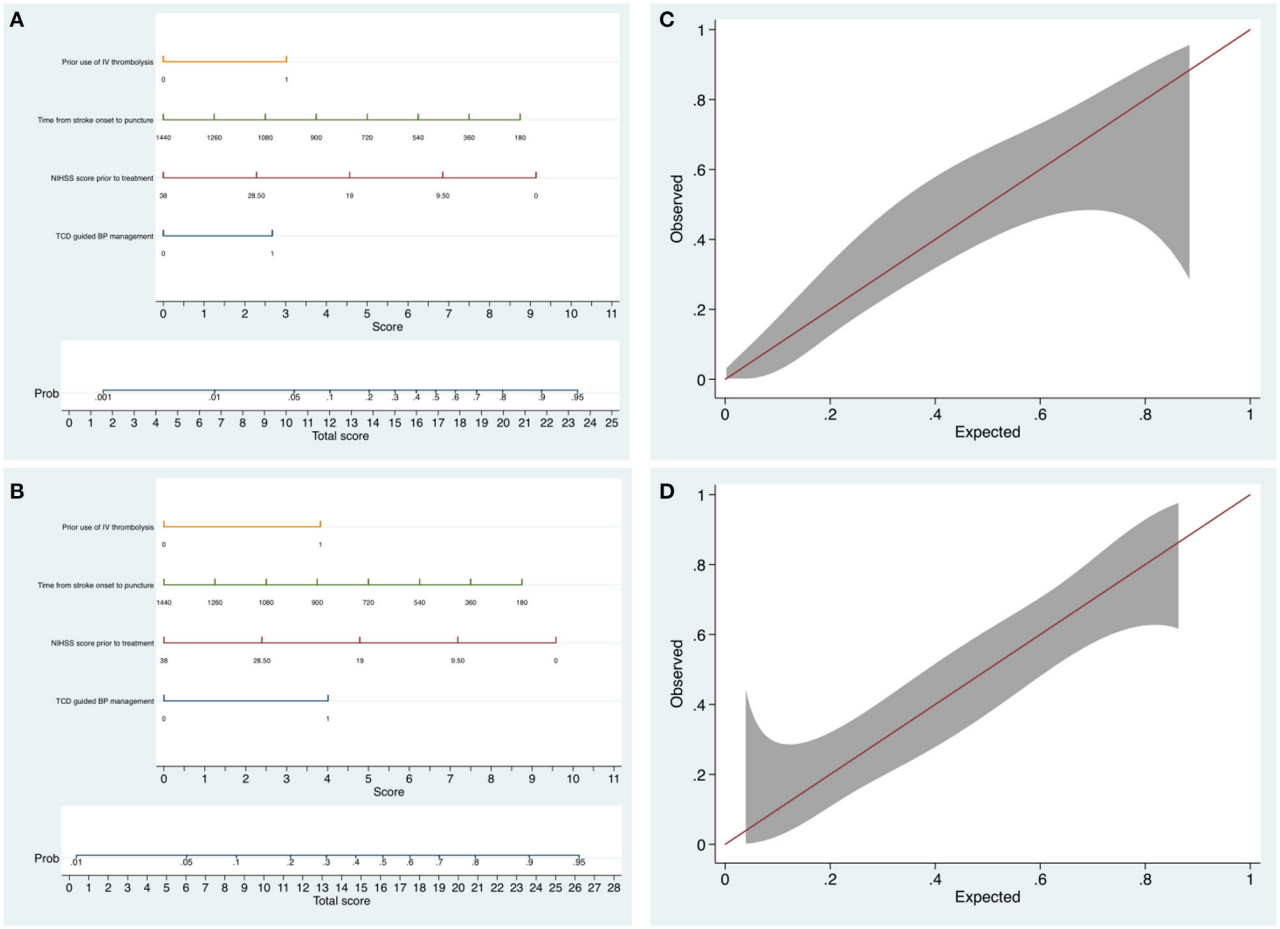
The nomograms and calibration plots for predicting the probability of excellent outcome and favorable outcome at 3 months. **(A)** The nomogram for predicting the probability of mRS 0–2. **(B)** The nomogram for predicting the probability of mRS 0–3. **(C)** The nomogram calibration plot for predicting the probability of mRS 0–2. Calibration plot displaying the observed proportion of patients who have functional independence in the PSM cohort with 95% CIs (vertical lines) against the predicted probability. **(D)** The nomogram calibration plot for predicting the probability of mRS 0–3.

No significant collinearity was observed for TCD use, baseline NIHSS, and OTP and IVT (variance inflation factor <1.2; condition index <8.6). Nomograms with all these significant predictors were constructed to show the risk stratification for excellent function outcome (Figure 4A) and favorable function outcome (Figure 4B). The calibration curves suggested that the nomograms were relatively well calibrated with satisfactory agreement between prediction and observation (Figures 4C,D). C-statistics of 0.77 (95% CI, 0.68–0.85) for the model predicting excellent outcome and 0.77 (95% CI, 0.69–0.86) for the model predicting favorable outcome indicated good model discriminative ability. The models were internally validated using 2,000 bootstrap samples to calculate the discrimination with accuracy of 0.77 (95% CI, 0.68–0.85) for the model predicting excellent functional outcome and 0.77 (95% CI, 0.69–0.86) for the model predicting favorable functional outcome, respectively.

## Discussion

In patients with acute ischemic stroke due to LVO who were eligible for treatment with EVT, TCD-guided BP management during the post-procedural 24-h period was associated with better functional outcomes at 3 months. This association is independent of demographics, vascular risk factors, admission SBP and DBP levels, baseline NIHSS scores and IVT, onset to groin puncture time, and reperfusion status.

We chose PSV ≥ 118 cm/s or iMFV/cMFV ≥ 125% as the sign of BP-lowering treatment, and PSV ≤ 74 cm/s as the sign of BP-raising treatment in this study. First, a previous ROC analysis has suggested that PSV ≥ 118 cm/s was an independent risk factor for early neurological deterioration, with a sensitivity of 88.1% and a specificity of 52.4%, respectively. Moreover, the risk of sICH and vasogenic cerebral edema also increased significantly when PSV ≥ 118 cm/s (14). Second, iMFV/cMFV ≥ 1.25 was chosen because Kneihsl et al. found that the risk of postinterventional ICH (OR 3.6, 95% CI 1.1 to 13.2) and mRS 3–6 at 90 days (OR 3.2, CI 1.1–9.7) increased significantly when MFV on the lesion side was higher than 125% that on the opposite side (9). Third, PSV ≤ 74 cm/s was chosen, because a previous RCT study has suggested that applying BP raising treatment in patients with PSV ≤ 74 cm/s significantly decreased the risk of early adverse prognosis (45.5 vs. 91.7%, *p* = 0.027) (12).

Around 50% of patients receiving EVT still had mRS >2 at 3 months, which left residual disability in a substantial number of patients. It is essential to investigate other modifiable factors that may improve clinical outcomes in patients with LVO. Few studies have examined the association between cerebral blood flow changes after EVT and long-term prognosis. Ischemic penumbra due to LVO stroke has impaired autoregulation and is more sensitive to BP changes (21). Monitoring 215 LVO stroke patients with bedside TCD, Kneihsl et al. found that 36% patients with successful angiographic reperfusion had abnormal cerebral blood flow, which was associated with a poor 90-day outcome (*p* = 0.004) (8). Meanwhile, patients with unsuccessful angiographic recanalization and normal sonographic MCA blood flow showed a higher probability of a favorable outcome compared to patients with abnormal MCA blood flow. Another research prospectively compared personalized, autoregulation-based BP targets with static systolic BP thresholds using a near-infrared spectroscopy-derived method (22). This study found that increased time with mean arterial pressure above the upper limit of autoregulation was associated with worse clinical outcomes at 90 days (OR 1.84, 95% CI 1.3–2.7, *p* = 0.002) and elevated rates of hemorrhagic transformation (10.9 vs. 16.0%, *p* = 0.042). Currently, the measurement instrument used mostly to monitor cerebral blood flow was MRI, which suffered from restrictions such as high technical effort and prohibition in unwell or unstable patients (23).

A tiered approach with lower BP targets was used when successful recanalization was achieved (2, 3). Current literatures supported that in patients with successful recanalization after EVT, controlling BP to a lower range can help improve the prognosis. However, most patients with LVO suffered from complicated vascular abnormalities, and generalized BP management schemes could not be applicable to all patients. A small study explored the impact of TCD regulation of hemodynamics on the prognosis of patients and found that BP controlling under TCD monitoring can help improve the prognosis. However, the small sample size (*n* = 90) and all conclusions were based on the subgroup analysis with patients divided into three groups which influenced the robustness of the results (12).

A borderline significant association between BP management and risk of symptomatic or any ICH was found in our study. The modest sample size and overall low rates of ICH may lead to this observed lack of significance. However, we noticed that the probability of symptomatic or any ICH decreased around 12% in patients in the TBM group. Elevated postinterventional BP and increased risk of ICH were observed in several retrospective studies (24, 25), and a significant association between abnormal cerebral blood flow by MRI and increased rates of hemorrhagic transformation was also observed (26). This finding is supported by the hypothesis that cerebral blood flow is a more direct method depicting the cerebral pressure-passive system.

In the present results, TCD use, baseline NIHSS, IVT, and OTP were risk factors related to prognosis at 3 months. Notably, this study indicated that use of IVT prior to EVT was a significantly protective factor for clinical outcomes at 3 months, which is in agreement with the recent meta-analysis (including 7,191 patients receiving bridge therapy and 4,891 patients receiving direct mechanical thrombectomy) showing that bridging with IVT led to better clinical outcomes (OR 1.43, 95% CI 1.28–1.61) (27). Nomograms have been used extensively in stoke research (28–31). With the combination of the factors easily available prior to and after the thrombectomy, the nomograms provide indications for early identification of patients' prognosis.

The strengths of our study lie in the prospective design and relatively intact data in the real world. There are some limitations in this research. Firstly, this study did not adjust for the collateral circulation status, so the relationship between BP management and prognosis in different collateral circulation status could not be investigated. It is reasonable to assume that BP management may lead to different effects for patients with poor collateral circulation. Secondly, our cohort study mainly consisted of patients with mTICI ≥2b; therefore, our results are mainly applicable to successfully recanalized patients. Research is needed to further evaluate the association between BP management and prognosis in non-recanalized patients. However, the low proportion of patients with mTICI <2b assisted in the reduction of the influence of a residual stenosis or other steno-occlusive processes on MCA blood flow measurements. The study population in this study is limited to Chinese, which may influence on the external validity of the results in other populations/ethnicities. Thirdly, a higher risk of selection bias could not be avoided due to the nonrandomized nature of this study, and a cautious interpretation of the results is urged. Moreover, occlusion site and stroke etiology were still significantly different between the NBM and TBM group after PSM. Fourthly, the data about periprocedural vasoactive drugs were not available in this study, which was significantly associated with the postoperative hemodynamics. We did the multivariable analysis with the adjustment for the baseline SBP, which might account for this effect to some extent.

The present study reported supporting evidence that BP management under TCD monitoring was independently associated with a higher odds of 3-month functional independence in AIS patients treated with EVT. Multicenter randomized controlled clinical trials with sufficient sample size are needed to truly establish the efficacy and safety of BP management under TCD monitoring in EVT-treated patients.

## Data Availability Statement

The raw data supporting the conclusions of this article will be made available by the authors, without undue reservation.

## Ethics Statement

The studies involving human participants were reviewed and approved by the Medical Ethics Committee of the First Affiliated Hospital of USTC. The patients/participants provided their written informed consent to participate in this study.

## Author Contributions

CT contributed to the study design, writing, analysis and interpretation of the data, literature search, and manuscript writing and editing. PX and YY contributed to the study design, study conduct, data analysis and interpretation, study reporting, and manuscript writing and editing. YZ, RL, and JL contributed to the statistical analysis and interpretation of data and revision of the manuscript. WH contributed to the study design, clinical conduct, analysis and interpretation of data, and editing of the manuscript and its approval for publication. All authors have approved the final version for submission.

## Funding

This study was funded by the Key Research and Development Projects of Anhui Province (No. 201904a07020086) and the Fundamental Research Funds for the Central Universities (wk9110000108). The study was designed, conducted, analyzed, and interpreted by the investigators independent of sponsors.

## Conflict of Interest

The authors declare that the research was conducted in the absence of any commercial or financial relationships that could be construed as a potential conflict of interest.

## Publisher's Note

All claims expressed in this article are solely those of the authors and do not necessarily represent those of their affiliated organizations, or those of the publisher, the editors and the reviewers. Any product that may be evaluated in this article, or claim that may be made by its manufacturer, is not guaranteed or endorsed by the publisher.

## References

[1] Saver JL Goyal M Bonafe A. Stent-retriever thrombectomy after intravenous t-PA vs. t-PA alone in stroke. N Eng J Med. (2015) 372:2285–95. 10.1056/NEJMoa1415061 25882376

[2] Jovin TG Chamorro A Cobo E. Thrombectomy within 8 hours after symptom onset in ischemic stroke. N Engl J Med. (2015) 372:2296–306. 10.1056/NEJMoa1503780 25882510

[3] Nogueira R Jadhav A Haussen D. Thrombectomy 6 to 24 Hours after Stroke with a Mismatch between Deficit and Infarct. N Engl J Med. (2018) 378:11–21. 10.1056/NEJMoa1706442 29129157

[4] Berkhemer O Fransen P Beumer D. A randomized trial of intraarterial treatment for acute ischemic stroke. N Engl J Med. (2015) 372:11–20. 10.1056/NEJMoa1411587 25517348

[5] Campbell BC Mitchell PJ Kleinig TJ. Endovascular therapy for ischemic stroke with perfusion-imaging selection. N Engl J Med. (2015) 372:1009–18. 10.1056/NEJMoa1414792 25671797

[6] Rodrigues FB Neves JB Caldeira D. Endovascular treatment versus medical care alone for ischaemic stroke: systematic review and meta-analysis. BMJ. (2016) 353:i1754. 10.1136/bmj.i1754 27091337 PMC4834754

[7] Rha J Saver J. The impact of recanalization on ischemic stroke outcome: a meta-analysis. Stroke. (2007) 38:967–73. 10.1161/01.Str.0000258112.14918.24 17272772

[8] Kneihsl M Niederkorn K Deutschmann H. Abnormal blood flow on transcranial duplex sonography predicts poor outcome after stroke thrombectomy. Stroke. (2018) 49:2780–82. 10.1161/strokeaha.118.023213 30355211

[9] Kneihsl M Niederkorn K Deutschmann H. Increased middle cerebral artery mean blood flow velocity index after stroke thrombectomy indicates increased risk for intracranial hemorrhage. J Neurointerv Surg. (2018) 10:882–87. 10.1136/neurintsurg-2017-013617 29288194

[10] Zhang Z Pu Y Mi D. Cerebral hemodynamic evaluation after cerebral recanalization therapy for acute ischemic stroke. Front Neurol. (2019) 10:719. 10.3389/fneur.2019.00719 31333570 PMC6618680

[11] Demchuk AM Burgin WS Christou I. Thrombolysis in brain ischemia (TIBI) transcranial Doppler flow grades predict clinical severity, early recovery, and mortality in patients treated with intravenous tissue plasminogen activator. Stroke. (2001) 32:89–93. 10.1161/01.str.32.1.89 11136920

[12] Chen H Su Y He Y. Controlling blood pressure under transcranial doppler guidance after endovascular treatment in patients with acute ischemic stroke. Cerebrovascular Dis (Basel, Switzerland). (2020) 49:160–69. 10.1159/000506855 32316014

[13] Chen Y Wang L Zhang J. Monitoring of patients with brainstem hemorrhage: A simultaneous study of quantitative electroencephalography and transcranial Doppler. Clin Neurophysiol. (2021) 132:946–52. 10.1016/j.clinph.2020.12.026 33636610

[14] He Y Su Y Rajah G. Trans-cranial Doppler predicts early neurologic deterioration in anterior circulation ischemic stroke after successful endovascular treatment. Chin Med J. (2020) 133:1655–61. 10.1097/cm9.0000000000000881 32604178 PMC7401737

[15] Hacke W Kaste M Fieschi C. Randomised double-blind placebo-controlled trial of thrombolytic therapy with intravenous alteplase in acute ischaemic stroke (ECASS II). Second European-Australasian Acute Stroke Study Investigators. Lancet (London, England). (1998) 352:1245–51. 10.1016/s0140-6736(98)08020-9 9788453

[16] Austin P. Balance diagnostics for comparing the distribution of baseline covariates between treatment groups in propensity-score matched samples. Stat Med. (2009) 28:3083–107. 10.1002/sim.3697 19757444 PMC3472075

[17] Austin P. An introduction to propensity score methods for reducing the effects of confounding in observational studies. Multivariate Behav Res. (2011) 46:399–424. 10.1080/00273171.2011.568786 21818162 PMC3144483

[18] Austin P A. comparison of 12 algorithms for matching on the propensity score. Stat Med. (2014) 33:1057–69. 10.1002/sim.6004 24123228 PMC4285163

[19] Austin PC. Optimal caliper widths for propensity-score matching when estimating differences in means and differences in proportions in observational studies. Pharm Stat. (2011) 10:150–61. 10.1002/pst.433 20925139 PMC3120982

[20] Cattaneo MD. Efficient semiparametric estimation of multi-valued treatment effects under ignorability. J Econom. (2010) 155:138–54. 10.1016/j.jeconom.2009.09.023

[21] Jusufovic M Sandset E Bath P. Effects of blood pressure lowering in patients with acute ischemic stroke and carotid artery stenosis. Int J Stroke. (2015) 10:354–9. 10.1111/ijs.12418 25472578

[22] Petersen N Silverman A Strander S. Fixed compared with autoregulation-oriented blood pressure thresholds after mechanical thrombectomy for ischemic stroke. Stroke. (2020) 51:914–21. 10.1161/strokeaha.119.026596 32078493 PMC7050651

[23] Yu S Liebeskind D Dua S. Postischemic hyperperfusion on arterial spin labeled perfusion MRI is linked to hemorrhagic transformation in stroke. J Cereb Blood Flow Metab. (2015) 35:630–7. 10.1038/jcbfm.2014.238 25564233 PMC4420881

[24] Butcher K Christensen S Parsons M. Postthrombolysis blood pressure elevation is associated with hemorrhagic transformation. Stroke. (2010) 41:72–7. 10.1161/strokeaha.109.563767 19926841

[25] Mistry E Mistry A Nakawah M. Systolic blood pressure within 24 hours after thrombectomy for acute ischemic stroke correlates with outcome. J Am Heart Association. (2017) 6:e006167. 10.1161/jaha.117.006167 28522673 PMC5524120

[26] Okazaki S Yamagami H Yoshimoto T. Cerebral hyperperfusion on arterial spin labeling MRI after reperfusion therapy is related to hemorrhagic transformation. J Cereb Blood Flow Metab. (2017) 37:3087–90. 10.1177/0271678x17718099 28665168 PMC5584703

[27] Wang Y Wu X Zhu C. Bridging thrombolysis achieved better outcomes than direct thrombectomy after large vessel occlusion: an updated meta-analysis. Stroke. (2021) 52:356–65. 10.1161/strokeaha.120.031477 33302795

[28] Yuan K Chen J Xu P. A Nomogram for predicting stroke recurrence among young adults. Stroke. (2020) 51:1865–67. 10.1161/strokeaha.120.029740 32390546

[29] Wu Y Chen H Liu X. A new nomogram for individualized prediction of the probability of hemorrhagic transformation after intravenous thrombolysis for ischemic stroke patients. BMC Neurol. (2020) 20:426. 10.1186/s12883-020-02002-w 33234113 PMC7685652

[30] Ma L Zhang S Li Z. Morbidity after symptomatic hemorrhage of cerebral cavernous malformation: a nomogram approach to risk assessment. Stroke. (2020) 51:2997–3006. 10.1161/strokeaha.120.029942 32951540

[31] Cappellari M Mangiafico S Saia V. IER-SICH Nomogram to predict symptomatic intracerebral hemorrhage after thrombectomy for stroke. Stroke. (2019) 50:909–16. 10.1161/strokeaha.118.023316 31233386

